# Co-occurring psychological distress and alcohol or other drug use among Indigenous Australians: Data from the National Aboriginal and Torres Strait Islander Health Survey

**DOI:** 10.1177/00048674241244601

**Published:** 2024-04-06

**Authors:** Breanne Hobden, Jamie Bryant, Robert Davis, Todd Heard, Jenn Rumbel, Jamie Newman, Bron Rose, David Lambkin, Rob Sanson-Fisher, Megan Freund

**Affiliations:** 1 Health Behaviour Research Collaborative, School of Medicine and Public Health, College of Health, Medicine, and Wellbeing, The University of Newcastle, Callaghan, NSW, Australia; 2 Equity in Health and Wellbeing Program, Hunter Medical Research Institute, New Lambton Heights, NSW, Australia; 3 Wiyiliin ta CAMHS, Hunter New England Local Health District, NSW Health, Newcastle, NSW, Australia; 4 Systems Neuroscience Group, Hunter Medical Research Institute, New Lambton Heights, NSW, Australia; 5 Wollotuka Institute, Purai Global Indigenous History Centre, The University of Newcastle, Callaghan, NSW, Australia; 6 Orange Aboriginal Medical Service, Orange, NSW, Australia; 7 Yimamulinbinkaan, Aboriginal Mental Health Service & Social Emotional Wellbeing Workforce, Hunter New England Mental Health Service, Hunter New England Local Health District, NSW Health, Newcastle, NSW, Australia; 8 Clinical Research Design and Statistics, Hunter Medical Research Institute, New Lambton Heights, NSW, Australia

**Keywords:** Aboriginal and Torres Strait Islander peoples, psychological distress, substance-related disorders, holistic health, comorbidity

## Abstract

**Objectives:**

To determine the prevalence and demographic, social and health characteristics associated with co-occurring psychological distress symptoms, risky alcohol and/or substance use among a national sample of Aboriginal and Torres Strait Islander people aged 15 years or older.

**Methods:**

This study uses secondary cross-sectional data from the 2018-19 National Aboriginal and Torres Strait Islander Health Survey (NATSIHS). Data were collected via face-to-face interviews with those living in private dwellings across Australia. Participants were Aboriginal and Torres Strait Islander people (*n* = 10,579) aged 15 years or older. Data pertaining to psychological distress, alcohol and substance use were obtained and weighted to represent the total population of Aboriginal and Torres Strait Islander people in Australia.

**Results:**

A total of 20.3% participants were found to have co-occurring psychological distress, risky alcohol use and/or substance use, and 4.0% reported co-occurrence of all three conditions. Female participants in a registered marriage and fully engaged in study or employment had lower rates of co-occurring conditions. Poorer self-rated health, one or more chronic conditions and increased experiences of unfair treatment and physical harm in the past 12 months were associated with increased rates of co-occurring conditions.

**Conclusion:**

A range of potential risk and protective factors were identified for co-occurring psychological distress, risky alcohol and/or substance use among Aboriginal and Torres Strait Islander people. This information is critical for planning effective holistic strategies to decrease the burden of suffering imposed upon the individual, family and community members impacted by co-occurring conditions.

## Introduction

Aboriginal and Torres Strait Islander people continue to demonstrate phenomenal collective strength and resilience throughout colonisation, including coping with intergenerational grief and trauma, systemic racism, dispossession and mental and physical health disparities (Dudgeon et al., 2010; Sun et al., 2012). The ongoing and devastating impacts of colonisation has resulted in Aboriginal and Torres Strait Islander people being disproportionately impacted by mental illness and alcohol and other drug (AOD) use (Australian Institute of Health and Welfare [AIHW], 2015). Mental health and AOD disorders contribute the largest chronic disease burden for Aboriginal and Torres Strait Islander people, representing 19% of disability-adjusted life years and 39% of years living with a disability (AIHW, 2016).

The co-occurrence (i.e. comorbidity) of mental illness and AOD misuse has been associated with exacerbated symptom severity, greater disability or impairment and increased risk of suicidal symptoms or acts compared with single conditions (Burns and Teesson, 2002; Quello et al., 2005). Australian population data suggest that 5.1% of individuals have comorbid anxiety, depressive or substance use disorders (Teesson et al., 2009). However, limited research has explored comorbidity of psychological distress and AOD misuse among Aboriginal and Torres Strait Islander people. The available research suggests that comorbidity is more prevalent among Aboriginal and Torres Strait Islander people than among non-Indigenous Australians. One study examined comorbidity of depression, anxiety and substance use disorders among 544 Aboriginal and Torres Strait Islander people attending Aboriginal Medical Services (AMS), the general community and two Aboriginal Land Council Reserves. This study found that 18.75% of the sample had a comorbid condition, representing a 3–4 fold higher rate than that of the general population (Nasir et al., 2018). A higher prevalence of comorbidity was reported for women than for men, and the AMS group had a higher prevalence than the Reserve group. However, this study was limited to a relatively small geographical region, with underrepresentation of remote/very remote populations.

For Aboriginal and Torres Strait Islander people, a number of risk factors are indicated to be associated with increased psychological distress including economic/social disadvantage, physical health problems and violence (Kelly et al., 2009). Furthermore, a recent systematic scoping review of racism and discrimination on the physical and mental health among Aboriginal and Torres Strait Islander people found that of the 10 studies examining overall mental health, all 10 reported racism to be associated with negative mental health (Kairuz et al., 2021). It also found three out of five studies found a negative association between racism and depression or anxiety (Kairuz et al., 2021). Conversely, social cohesion, connection to land and culture and a sense of wellbeing have been highlighted as protective factors for social and emotional wellbeing (Kelly et al., 2009). Research undertaken in the general Australian population has found that being younger, male, in an unstable relationship, unemployed and diagnosed with at least one physical illness were associated with increased comorbid mental health and/or AOD use (Burns et al., 2001).

No studies have examined a broad range of demographic, social or health characteristics associated with comorbid mental illness and AOD use among Aboriginal and Torres Strait Islander Peoples. Such an examination may identify at-risk groups, as well as risk and protective factors that may influence comorbidity (Procter, 2005). Given the significant burden of disease from mental illness and AOD use among Aboriginal and Torres Strait Islander Peoples (Australian Bureau of Statistics, 2013), further comprehensive studies are needed to guide policy and healthcare delivery.

## Aims

The purpose of this study is to determine, among a national sample of Aboriginal and Torres Strait Islander people aged 15 years or older, the (1) prevalence of and (2) demographic, social and health characteristics associated with self-reported co-occurring psychological distress symptoms, risky alcohol and/or substance use.

## Methods

### Community priority framework

This research was conceptualised to align with action areas in the ‘National Strategic Framework for Aboriginal and Torres Strait Islander Peoples’ Mental Health and Social and Emotional Wellbeing’ (Australian Government, 2017). This framework was built upon consultation with over 30 Aboriginal and Torres Strait Islander communities, as well as more than 180 organisations, service providers and expert groups. This research aligns with the following action area: ‘*Aboriginal and Torres Strait Islander communities and cultures are strong and support social and emotional wellbeing and mental health*’ (Outcome 2.1).

### Indigenous leadership for the study

This study concept arose through a collaboration between Indigenous and non-Indigenous researchers and those working in the health field on an Australian Rotary Health Fellowship to understand the co-occurrence of mental health conditions and substance use among Aboriginal and Torres Strait Islander Peoples. Co-authors JR, TH, RD, JN and BR are Aboriginal members of the team and formed the Aboriginal Advisory Group for this project. In addition to lived experiences, the Aboriginal Advisory Group has expertise in mental health, substance use and health services. The remaining authors are non-Indigenous. The aims of the study were discussed and revised through ongoing collaborative discussion with the whole research team. Inclusion of study variables and interpretation of results were iterated and reviewed by Indigenous and non-Indigenous team members throughout the project. For example, the variables exploring experiences of racism and violence were included based on recommendation by members of the Aboriginal Advisory Group.

### Data source

Data were drawn from the 2018-19 National Aboriginal and Torres Strait Islander Health Survey (NATSIHS) (Australian Bureau of Statistics, 2019). Governance and custodianship of these data are held with the Australian Bureau of Statistics (ABS), which is Australia’s national statistical agency. The ABS adheres to strict privacy protocols, including de-identifying the data and applying various confidentiality processes (including perturbation) to ensure a person cannot be identified in the data. Access to the data by third parties is strictly controlled.

### Ethics

Ethics approval was granted by Aboriginal Health and Medical Research Council (AH&MRC) Human Research Ethics Committee (1802/21).

### Eligibility

The NATSIHS survey included Aboriginal and Torres Strait Islander people living in private dwellings, in non-remote and remote areas, including sampling from discrete Indigenous communities. Those excluded were (1) visitors to private dwellings staying for <6 months; (2) households where all residents were <18 years old; (3) those living in non-private dwellings (e.g. nursing homes, hospitals); (4) boarding school students; (5) non-Australian diplomats or defence forces and their households and (6) overseas visitors.

### Consent

Survey participants were informed by the interviewer of the ABS Privacy Policy and how personal information collected for the purpose of production of official statistics is handled, consistent with the Privacy Act 1988 (Privacy Act) and the Australian Privacy Principles. Participants are also informed of the ABS’ authority to collect, use and release statistical information (Census and Statistics Act 1905). Following this, the participant provides verbal consent to undertake the interview.

### Data collection

Data were collected face-to-face by trained interviewers and input into a computer-based questionnaire. Interviewers received training from the ABS in cultural awareness, as well as completing training and exercises in understanding the survey procedures and content. Where possible, local Aboriginal and Torres Strait Islander advisors assisted in conducting interviews. During data collection, one household member (aged ⩾18 years) was asked to provide basic demographic details for all household members and answer general household questions. Up to two adults (⩾18 years) and two children (<18 years) per household were randomly selected from non-remote areas and one adult and one child per household from remote areas. Where possible, personal interviews were then conducted with household members who were older than 15 years.

### Measures

The following measures were used:

*Self-reported psychological distress symptoms*: The Kessler 5 (K5) measure was used to examine recent symptoms of non-specific psychological distress. This tool has strong psychometric properties and has been demonstrated to be acceptable, culturally appropriate and valid for use among a national sample of Aboriginal and Torres Strait Island people (Brinckley et al., 2021). Scores of 12 or higher indicate high levels of distress and a probable mental health diagnosis (Brinckley et al., 2021).
*Self-reported alcohol risk levels*: NATSIHS items were extracted to align with current Australian alcohol guidelines (National Health and Medical Research Council, 2020). These guidelines state: ‘. . . *healthy men and women should drink no more than 10 standard drinks a week and no more than 4 standard drinks on any one day*’. Therefore, lifetime risk included participants who indicated having three or more standard drinks at least 3 days per week in the last 12 months. While this estimation would have captured those reporting nine standard drinks per week, it was not possible to align this figure exactly to the included items. Consuming five or more standard drinks in a day, at least 13 times in the last 12 months (i.e. approx. once every 4 weeks or more) was considered binge drinking. Those who met either lifetime or binge drinking risk criteria were considered ‘at-risk’.
*Self-reported substance use*: Participants were asked if they used any of the following in the last 12 months: petrol sniffing; amphetamines, ice or speed; cocaine; heroin; kava; lysergic acid diethylamide (LSD) or synthetic hallucinogens; marijuana, hashish or cannabis resin; pain killers or analgesics for non-medical purposes; tranquilisers or sleeping pills for non-medical purposes; ecstasy or designer drugs; methadone for non-medical purposes; naturally occurring hallucinogens; other inhalants and other substances. Any use of these substance in the past 12 months was considered ‘at-risk’.
*Sociodemographic characteristics*: Several demographic, social and health variables were selected based on an examination of important characteristics identified in the literature and guidance from the study’s Aboriginal Reference Group on important cultural and social factors. Demographic variables included the persons: age; sex; marital status; household postcode (to derive Socio-Economic Indexes for Areas [SEIFA]); and employment/current study. Health variables included self-assessed health status (responses: excellent; very good; good; fair; poor) and current chronic conditions. Social and cultural variables included satisfaction with own knowledge of culture; whether proud of culture/being Aboriginal or Torres Strait Islander; identification with a tribal group, language group or clan; experiences of physical harm, as a victim, in the last 12 months, and frequency of unfair treatment due to being Aboriginal and Torres Strait Islander (i.e. experiences of racism).

### Data analysis

The data were analysed in RStudio v1.4.1717. All analyses were weighted using individual participant weights and 250 replicate weights using the jackknife method. Descriptive statistics are presented as weighted frequency, weighted percentages and weight percentage standard error. A SEIFA score was calculated to determine the socio-economic conditions of the participants’ geographic region, with scores of 1–5 indicating economic disadvantage and scores ⩾6 indicating economic advantage. An alpha level of 0.05 was specified for all tests and confidence intervals (CI). The factors associated with the presence of comorbid conditions were examined using logistic regression (crude and adjusted models). For the regression, presence of any combination of the self-reported conditions (psychological distress, alcohol use, substance use) versus no conditions/single conditions were used and are presented as odds ratios (OR; 95% CI) with *p*-values. ‘Not applicable’ was a variable collated by the ABS, and these responses were excluded from the regression analyses. The Rao-Scott likelihood Ratio Test was used to examine the overall variable’s *p*-value, and each category was compared to the reference group using pairwise comparisons.

## Results

Overall, 73.4% of selected households had at least one person complete the survey. Data from 10,579 individuals were extrapolated and weighted to represent the total Aboriginal and Torres Strait Islander population in Australia. Most of the participants were aged 15–34 years (51%), were female (51.7%) and were located in an area of economic disadvantage (81.4%). The full participant characteristics can be found in Table 1.

**Table 1.** Weighted estimates of the population percentages for demographic data.

Characteristic	Subgroup	%	% SE
Demographic variables			
Age	15–24	29.0	0.00
	25–34	22.0	0.00
	35–44	15.6	0.00
	45–54	15.2	0.00
	55–64	10.7	0.00
	65+	7.4	0.00
Sex	Female	51.7	0.00
	Male	48.3	0.00
Social marital status	Registered marriage	20.0	0.97
	Defacto marriage	16.0	0.84
	Not married	64.1	1.26
SEIFA	1–5	81.4	1.51
	6–10	18.6	1.51
Employment/current study	Fully engaged	39.7	1.19
	Partially engaged	15.1	0.88
	No study or employment	45.2	1.16
Health variables			
Self-assessed health	Excellent	16.1	0.86
	Very good	28.5	1.20
	Good	31.5	1.17
	Fair	15.3	0.76
	Poor	8.6	0.65
Current chronic conditions	0	42.9	1.16
	1 or more	57.1	1.16
Social and cultural variables			
Satisfaction with own knowledge of culture	Very satisfied	21.1	1.17
	Satisfied	28.1	1.13
	Neutral	18.1	1.09
	Not very satisfied	18.0	0.99
	Not at all satisfied	4.4	0.54
	Not applicable	10.3	0.22
Identifies with tribal group, language group or clan	Yes	58.6	1.31
	No	30.8	1.31
	Not applicable	10.5	0.23
Whether proud of culture/being Aboriginal or Torres Strait Islander	Yes (proud)	87.2	0.49
	No (not proud)	2.5	0.43
	Not applicable	10.3	0.22
Experience of physical harm in last 12 months	Yes	5.8	0.55
	No	85.7	0.70
	Not stated	0.7	0.14
	Not applicable	7.9	0.48
Frequency of unfair treatment in last 12 months due to being Aboriginal and Torres Strait Islander	Always	1.4	0.34
	Often	2.8	0.36
	Sometimes	7.5	0.56
	Rarely	7.2	0.63
	Only once	2.7	0.37
	None	65.3	1.19
	Does not know	6.0	0.79
	Not applicable	7.1	0.45

SE = standard error.

### The prevalence of co-occurring conditions

**Figure 1.**
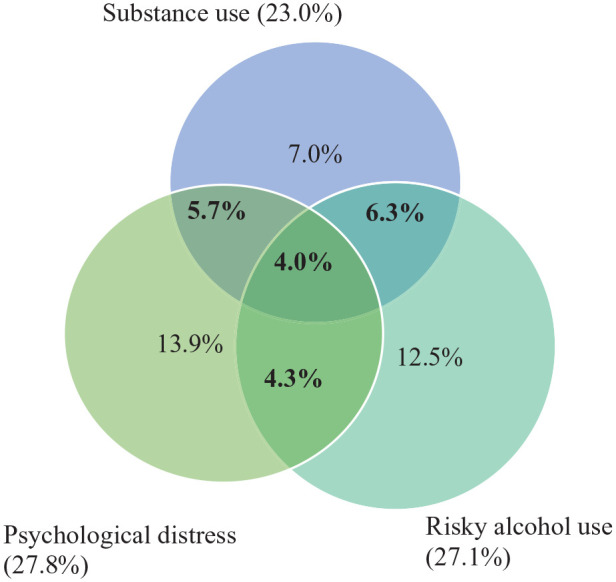
Prevalence of psychological distress, risky alcohol use and risky substance use and their comorbidity among Aboriginal and Torres Strait Islander people. Bold values = comorbid proportions.

A total of 20.3% participants met the criteria for co-occurring psychological distress, risky alcohol use and/or substance use, and 4.0% met all three criteria. The full list of conditions can be found in Table 2 and Figure 1.

**Table 2.** Weighted estimates of the population percentages for self-reported psychological distress (Kessler 5), risky alcohol use and substance use.

	Subgroup	%	% SE
Single conditions			
Psychological distress (Kessler 5)	Low/moderate (5–11)	60.1	1.05
	High/very high (12–25)	27.8	1.12
	Not applicable	12.1	0.40
Alcohol risk category	Binge risk only	17.6	1.02
	Lifetime risk only	1.2	0.25
	Lifetime and binge risk	8.2	0.70
	No risk	70.5	1.20
	Not applicable	2.4	0.32
Any substance use (for non-medical use) in the past 12 months	Yes	23.0	1.05
	No	56.6	1.19
	Not applicable	20.3	1.01
Comorbid conditions			
Psychological distress and risky alcohol use	Yes	4.3	0.49
Psychological distress and substance use	Yes	5.7	0.57
Risky alcohol use and substance use	Yes	6.3	0.65
Psychological distress, risky alcohol use and substance use	Yes	4.0	0.44
Total for any comorbid conditions		20.3	0.99

SE = standard error.

### Characteristics associated with increased rates of co-occurring conditions

The full logistic regression model can be found in Table 3. Older participants reported co-occurring conditions less often than participants in the 15- to 24-year-old age group (*p* ⩽ 0.001). Males self-reported co-occurring conditions more than twice as often as females (OR = 2.43, 95% CI [1.88, 3.14], *p* ⩽ 0.001). Participants who were in a registered marriage reported co-occurring conditions less often than participants who were not married or those in a de facto marriage (*p* = 0.011). Participants who were fully engaged with study or employment reported co-occurring conditions less often than participants who were not (*p* = 0.028).

**Table 3.** Sociodemographic characteristics associated with the presence of comorbid conditions vs single or no condition.

Characteristic	Subgroup	Crude		Adjusted	
		OR (95% CI)	*p* Value	OR (95% CI)	*p* Value
Age	15–24	Reference	**<0.001** ^a^	Reference	**<0.001** ^a^
	25–34	1.23 (0.86, 1.75)	0.258	1.09 (0.71, 1.65)	0.697
	35–44	0.84 (0.60, 1.20)	0.340	0.69 (0.46, 1.02)	0.065
	45–54	1.06 (0.75, 1.51)	0.751	0.81 (0.51, 1.27)	0.352
	55–64	0.87 (0.58, 1.29)	0.484	0.53 (0.32, 0.88)	0.014
	65+	0.19 (0.10, 0.36)	<0.001	0.12 (0.06, 0.22)	<0.001
Sex	Female	Reference	**<0.001** ^a^	Reference	**<0.001** ^a^
	Male	1.95 (1.57, 2.43)	<0.001	2.43 (1.88, 3.14)	<0.001
Social marital status	Registered marriage	Reference	**<0.001** ^a^	Reference	**0.011** ^a^
	Defacto marriage	2.31 (1.32, 4.03)	0.003	2.09 (1.16, 3.77)	0.014
	Not married	2.69 (1.78, 4.05)	<0.001	2.02 (1.27, 3.22)	0.003
SEIFA	1–5	Reference	**0.624**	Reference	**0.832**
	6–10	0.92 (0.64, 1.31)	0.630	1.05 (0.67, 1.65)	0.838
Employment/current study	Fully engaged	Reference	**0.068**	Reference	**0.028** ^a^
	Partially engaged	1.23 (0.82, 1.85)	0.311	1.48 (0.91, 2.38)	0.110
	Not engaged	1.40 (1.05, 1.86)	0.021	1.61 (1.13, 2.29)	0.008
Self-assessed health	Excellent/Very good	Reference	**<0.001** ^a^	Reference	**<0.001** ^a^
	Good	1.57 (1.19, 2.07)	0.002	1.47 (1.08, 2.01)	0.015
	Fair/Poor	2.34 (1.75, 3.14)	<0.001	2.49 (1.71, 3.62)	<0.001
Number of chronic conditions	0	Reference	**0.004** ^a^	Reference	**0.044** ^a^
	1 or more	1.39 (1.11, 1.74)	0.004	1.34 (1.01, 1.78)	0.043
Satisfaction with own knowledge of culture	Very satisfied/Satisfied	Reference	**0.099**	Reference	**0.338**
	Neutral	1.13 (0.81, 1.57)	0.467	1.06 (0.71, 1.57)	0.775
	Not very/Not at all satisfied	1.39 (1.05, 1.84)	0.022	1.30 (0.93, 1.81)	0.123
Identifies with tribal group, language group or clan	Yes	Reference	**0.850**	Reference	**0.794**
	No	1.02 (0.80, 1.31)	0.856	1.04 (0.77, 1.40)	0.801
Proud of culture/being Aboriginal or Torres Strait Islander	Yes (proud)	Reference	**0.487**	Reference	**0.603**
	No (not proud)	1.43 (0.53, 3.86)	0.479	0.83 (0.40, 1.71)	0.612
Experience of physical harm in last 12 months	Yes	2.73 (1.84, 4.05)	<0.001	1.95 (1.23, 3.09)	0.005
	No	Reference	**<0.001** ^a^	Reference	**0.006** ^a^
Frequency of unfair treatment in the last 12 months	None	Reference	**<0.001** ^a^	Reference	**0.001** ^a^
	Rarely/Only once	1.56 (1.05, 2.33)	0.028	1.48 (0.96, 2.30	0.077
	Sometimes	1.85 (1.32, 2.59)	<0.001	1.54 (1.07, 2.21)	0.019
	Always/Often	3.09 (1.87, 5.10)	<0.001	2.96 (1.69, 5.18)	<0.001

Bolded values = overall *p*-value for each characteristic using Rao-Scott Likelihood Ratio Test.

a Significant overall *p*-value.

Participants who had better self-assessed health reported co-occurring conditions less often than participants who had poorer self-assessed health (*p* ⩽ 0.001). Participants with one or more chronic conditions reported co-occurring conditions more often than participants with no chronic conditions (OR = 1.34, 95% CI [1.01, 1.78], *p* = 0.043).

There were no significant findings for the cultural factors explored (satisfaction with own knowledge of culture; identifying with tribal group, language group or clan and whether proud of culture/being Aboriginal or Torres Strait Islander). Participants who had experienced physical harm in the last 12 months were almost twice as likely to report co-occurring conditions compared to participants who had not (OR = 1.95, 95% CI [1.23, 3.09], *p* = 0.005). There was an overall trend for participants who had reported more unfair treatment in the last 12 months to self-report co-occurring conditions more than participants who had not experienced unfair treatment (i.e. racism) in the last 12 months (*p* = 0.001).

## Discussion

This study provides the first exploration of co-occurring psychological distress and AOD misuse among a nationally representative sample of Aboriginal and Torres Strait Islander people. The findings indicate that approximately 1 in 5 Aboriginal and Torres Strait Islander people experience two or more of these conditions. While the rates of comorbid conditions were similar across the different combinations of conditions, the highest rate of comorbidity was found for co-occurring risky alcohol and substance use (6.3%). Approximately 1 in 25 individuals experienced co-occurring risky alcohol, substance use and psychological distress. Although somewhat outdated, 2007 Australian general population data found the prevalence of diagnoses of two or more anxiety, affective or substance use disorders to be 5.1% (Teesson et al., 2009). The high proportion of co-occurring conditions (20.3%) in this study aligns with previous research suggesting higher rates among Aboriginal and Torres Strait Islander people than among non-Indigenous people (Nasir et al., 2018).

Co-occurring conditions were more common among those who had been a victim of physical harm in the last 12 months. Those with perceived poorer self-assessed health and those with one or more chronic conditions also reported higher rates of co-occurring conditions. While it is not possible to explore a causal relationship between these characteristics in this study, these findings are useful for informing potential future support strategies among Aboriginal and Torres Strait Islander people experiencing these co-occurring conditions. For example, the provision of holistic support is likely to be beneficial for those who report physical harm either in a health or legal setting. This could occur through conducting screening and referrals for psychological distress and AOD use, in addition to addressing physical harm.

Co-occurring conditions were also more common among those who perceived experiencing more unfair treatment in the last 12 months. This finding aligns with previous literature demonstrating a link between experiences of discrimination and mental health outcomes (Kairuz et al., 2021; Pascoe and Smart Richman, 2009). It is important to consider that, in addition to being associated with poorer outcomes, perceptions of unfair treatment may deter an individual from accessing the healthcare system or other support services for their mental health or substance use conditions (Heard et al., 2022). The increased risk of co-occurring psychological distress and AOD use among those who experienced more unfair treatment due to being Aboriginal and Torres Strait Islander highlights that the strategies for harm minimisation should account for the wider social context in which Aboriginal and Torres Strait Islander people exist. In 2018, the Australian Medical Association included interpersonal and institutional racism as an important target for rebuilding the Close the Gap health strategy (Johansen et al., 2018). This work further highlights a need for priority public health strategies to address systemic injustice and promote recognition and celebration of Aboriginal and Torres Strait Islander Peoples as the First Peoples of this land and the oldest living culture.

There were several demographic variables associated with comorbidity, including being younger, male, unmarried and not engaged in study or employment. These findings align with previous population data exploring characteristics associated with comorbidity of depressive, anxiety and substance use disorders (Teesson et al., 2009). Being in a registered marriage appeared to be a protective factor, aligning with data from the 2008 National Aboriginal and Torres Strait Islander Social Survey (NATSISS), indicating married people had significantly less psychological stress and better mental health (Dockery, 2011). It is unclear why being in a de facto marriage did not include the same protective impact as a registered marriage. Employment is commonly associated with being a protective factor for mental health and AOD use (Modini et al., 2016; Stone et al., 2012). The need for whole of person understanding and care is further emphasised by the number of demographic factors associated with increased comorbidity.

### Limitations

The findings should be considered in light of several limitations. The NATSIHS used screening tools to assess psychological distress and AOD use. A standardised measure with validated cut points was used for the K5; however, for alcohol use, survey items needed to be aligned with the national Australian alcohol guidelines (NHMRC, 2020) for analysis. While a clinical diagnosis represents the gold standard for prevalence, sub-clinical levels may still impact the quality of life and functioning of individuals (Fehm et al., 2008; Rai et al., 2010; Roberts et al., 2015), particularly for comorbidity as the cumulative impact of these symptoms may cause significant burden without necessarily meeting diagnostic thresholds for a single condition (Lewinsohn et al., 2004; Shankman et al., 2009). The ABS also acknowledge that substance use was likely to be underreported as these questions were voluntary, and the sensitive nature of the items may have impacted willingness to respond. This may have occurred more frequently among remote areas as individuals provided responses directly to the interviewer and other household members may have been present. The study was limited to the factors that were included in the NATSIHS survey, and therefore, other important factors that may be associated with comorbidity (e.g. family history) could not be explored. Combining single conditions with no conditions may have obscured the contribution of these individual factors. While it was beyond the scope of the study to examine associated variables for AOD use only, psychological distress only and their co-occurrence, preliminary data analysis suggested that all the significant variables in the presented regression were also significant when comparing those with co-occurring conditions to those with no conditions (data not shown).

### Future directions

To understand and reduce the high proportion of comorbid psychological distress and AOD misuse, there is a need for greater research and government resource investment in this area. Research to examine strategies to decrease and treat comorbidity should then be explored, with Aboriginal communities and organisations leading the direction of such strategies. Importantly, researchers, healthcare providers and stakeholders need to ensure a clear commitment to integrated and holistic health. Efforts should be undertaken to consider how systems that work in silo, such as general and specialised healthcare, the legal system and social welfare, can work together to achieve improved outcomes for Aboriginal and Torres Strait Islander Peoples.

## Conclusion

This study indicates a high prevalence of co-occurring psychological distress and AOD misuse with a wide range of risk and protective factors was associated with psychological distress and AOD comorbidity for Aboriginal and Torres Strait Islander people. This information is critical for planning effective holistic strategies to decrease the burden of suffering imposed upon the individual, family and community members impacted by comorbidity.
